# Nucleosome Positioning

**DOI:** 10.5402/2012/245706

**Published:** 2012-10-15

**Authors:** Hiromi Nishida

**Affiliations:** Agricultural Bioinformatics Research Unit, Graduate School of Agricultural and Life Sciences, University of Tokyo, Tokyo 113-8657, Japan

## Abstract

Nucleosome positioning is not only related to genomic DNA compaction but also to other biological functions. After the chromatin is digested by micrococcal nuclease, nucleosomal (nucleosome-bound) DNA fragments can be sequenced and mapped on the genomic DNA sequence. Due to the development of modern DNA sequencing technology, genome-wide nucleosome mapping has been performed in a wide range of eukaryotic species. Comparative analyses of the nucleosome positions have revealed that the nucleosome is more frequently formed in exonic than intronic regions, and that most of transcription start and translation (or transcription) end sites are located in nucleosome linker DNA regions, indicating that nucleosome positioning influences transcription initiation, transcription termination, and gene splicing. In addition, nucleosomal DNA contains guanine and cytosine (G + C)-rich sequences and a high level of cytosine methylation. Thus, the nucleosome positioning system has been conserved during eukaryotic evolution.

## 1. Introduction

Eukaryotic genomic DNA is packaged with histone proteins to form chromatin [1, 2]. The most fundamental repeating unit of chromatin is the nucleosome, which consists of an octamer of histones (2 copies of each histone protein: H2A, H2B, H3, and H4) and the genomic DNA wrapped around the octamer [3, 4]. Modification (e.g., acetylation, methylation, and phosphorylation) of the nucleosomal core histones influences chromatin structure and biological functions [5–7]. The modified nucleosome should be formed at the genomic position or in the genomic region. In this paper, I will focus on nucleosome positioning (not histone modification), because nucleosome positioning is not only related to compacting the genomic DNA but also to gene regulation [8–17]. 

Due to the development of DNA sequencing technology and genomic tiling array technology, genome-wide nucleosome mapping has been performed in a wide range of eukaryotic species, including the budding ascomycetous yeast, *Saccharomyces cerevisiae* [19]; the nematode, *Caenorhabditis elegans* [20]; the fruit fly, *Drosophila melanogaster* [21]; humans, *Homo sapiens* [22]; the malaria parasite, *Plasmodium falciparum* [23]; the filamentous ascomycete, *Aspergillus fumigatus* [24]; the fission ascomycetous yeast, *Schizosaccharomyces pombe* [25]; the plant, *Arabidopsis thaliana* [26]; several ascomycetous yeasts [27]; the mouse, *Mus musculus* [28]; the basidiomycete, *Mixia osmundae* [29]; the amoebozoa, *Dictyostelium discoideum* [30]. 

## 2. Nucleosome Positioning and DNA Sequence Preference

The DNA sequence plays an important role in nucleosome positioning [31–37]. Genome-wide analyses of nucleosome positioning have revealed that DNA sequence preference exists for nucleosome occupancy [29, 38, 39]. The nucleosome occupancy reflects average nucleosome positioning levels on a given region of DNA in a population of cells [40–43]. For example, the dinucleotide sequences AA and TT are depleted in nucleosome-forming regions in different organisms [29, 39, 44], whereas the G + C content is highly correlated with nucleosome occupancy [45, 46]. In addition, it has been reported that nucleosomal DNA cytosines are more highly methylated than nucleosome linker DNA cytosines in humans and the plant *Arabidopsis* [26]. These results suggest that DNA sequence preference in nucleosome occupancy has been conserved during eukaryotic evolution. 

**Figure 1:**
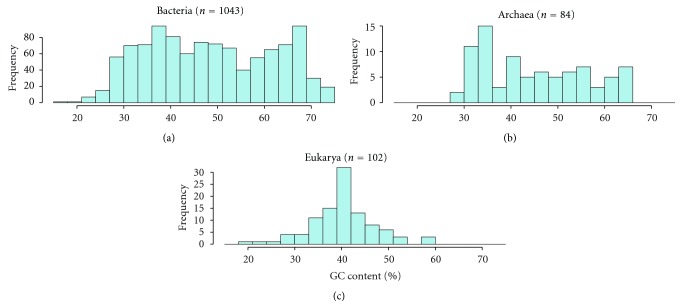
Distribution of the genomic G + C content of Bacteria, Archaea, and Eukarya. The G + C content data were obtained from the Genome Composition Database [18].

Genome-wide nucleosome positioning data suggest that nucleosome occupancy restricts the range of genomic G + C content. Bacteria and Archaea, which lack nucleosomes, have a wide range of G + C content. In contrast, the genomic G + C content distribution of Eukarya is completely different from that of Bacteria and Archaea (Figure 1). This distribution difference may be related to the differences in the conservation level of histones and nucleoid-associated proteins; although histone proteins are highly conserved between different organisms, nucleoid-associated proteins vary among Bacteria and Archaea [47–50]. 

## 3. Nucleosome Positioning around the Transcription Start Site

Nucleosome depletion in the vicinity of the transcription start site (TSS) has been indicated [51–53]. Indeed, nucleosome-free regions are pervasive in the gene promoters of yeast [26, 54, 55]. Moreover, the nucleosome organization around TSSs is very similar among different organisms [20, 29, 30, 39, 54, 55]. The nucleosome position profile is sharper in the downstream region of the TSS. Nucleosomes downstream from the nucleosome-free region are well positioned, with positioning decaying with increasing distance into the protein-coding region. Nucleosome positioning is more conserved in gene promoters than in gene bodies, suggesting that nucleosome positioning in the gene promoter plays an important role in gene transcription [19, 27, 52, 56, 57]. 

On the other hand, nucleosome positioning *in vivo* differs from that *in vitro*, indicating that systems other than DNA sequence preference are involved in nucleosome positioning [40, 41, 58]. Recently, it was reported that the most conserved nucleosome position (the +1 nucleosome), which is the sharpest in the nucleosome position profile, is maintained by ATP-dependent factors in *S. cerevisiae* [59, 60]. It remains uncertain whether nucleosome positioning in the gene promoter has been evolutionarily conserved as a major driving force in gene expression [15, 27, 36] or not [57, 61, 62].

## 4. Nucleosome Positioning around the Translation (or Transcription) End Site

Genome-wide nucleosome mapping analyses of the ascomycete *S. cerevisiae* revealed that nucleosome depletion is also found around translation end sites as well as TSSs [63, 64]. In the basidiomycete *M. osmundae*, dinucleosome—but not mononucleosome—depletion is clearly found around TSSs and translation end sites [29]. These results suggest that the nucleosome linker DNA length of *M. osmundae* around TSSs and translation end sites is shorter than that of *S. cerevisiae*. Nucleosome depletion around transcription end sites is also found in *Drosophila* and *Dictyostelium* [21, 30]. The regions around both transcription start and end sites have DNA sequences rich in adenine and thymine, which disfavor core histones [21, 30, 54]. Recently, some chromatin remodelers have been reported to locate around transcription start and end sites in *S. cerevisiae* [65]. 

## 5. Nucleosome Positioning in Exonic and Intronic Regions

Chromatin structure may be linked to gene splicing [66, 67]. Genome-wide nucleosome mapping analyses have shown that the nucleosome occupancy level in exons is higher than that in introns [68–72]. DNA sequence differences between exons and introns are correlated with nucleosomal DNA preferences [73], as exon DNA sequences have a higher G + C content than intron DNA sequences [70]. As described above, nucleosomal DNA prefers (G + C)-rich sequences. 

## 6. Conclusions

**Figure 2:**
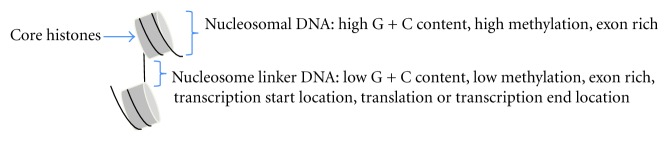
Difference between nucleosome-forming and linker regions.

Although the nucleosome positioning system differs between the ascomycetous budding yeast *S. cerevisiae* and the ascomycetous fission yeast *Sch. pombe* [25], genome-wide comparative analyses of nucleosome positions have revealed that nucleosome positioning shares a common feature among different organisms. Nucleosomal DNA has a higher G + C content and a higher level of cytosine methylation than nucleosome linker DNA (Figure 2). In addition, nucleosome positioning is found more frequently in exonic than in intronic regions. Transcription start sites and translation (or transcription) end sites are more frequently located in nucleosome linker DNA than in nucleosomal DNA. Thus, not only the structures of core histone proteins but also the nucleosome positioning systems have been greatly conserved during eukaryotic evolution.
